# The Effect of Calcium Sulfate on the Hydration and Properties of Red Mud-Based Calcium Ferroaluminate Cement Clinker

**DOI:** 10.3390/ma17205064

**Published:** 2024-10-17

**Authors:** Nan Shi, Ya Ma, Xiang Zhang, Jun Li, Xiaolei Lu, Lina Zhang, Xin Cheng

**Affiliations:** 1 Shandong Hi-Speed Construction Management Group Co., Ltd., Jinan 250001, China; 0531shinan@163.com (N.S.); zjiangly@163.com (Y.M.); forzaapis@126.com (J.L.); 2 Shandong Hi-Speed Peninsula Investment Co., Ltd., Yantai 264119, China; 3 Shandong Provincial Key Laboratory of Preparation and Measurement of Building Materials, University of Jinan, Jinan 250022, China; 202411100030@stu.ujn.edu.cn (X.Z.); mse_zhangln@ujn.edu.cn (L.Z.); chengxin@ujn.edu.cn (X.C.)

**Keywords:** red mud-based ferroaluminate cement, calcium sulfate type, hydration, mechanical properties

## Abstract

The hydration of high-alkali red mud-based ferroaluminate cement (RCFA) clinker with calcium sulfate needs to be regulated. This study explored the effects of the calcium sulfate type and dosage on the hydration and properties of high-alkali RCFA clinker. The research results show that when 4% gypsum was added, the 3 d compressive strength of cement was 39.1 MPa, and the 28 d compressive strength was 63.2 MPa. The 28 d strength increased by 61.6% compared with the 3 d strength. The properties of cement paste can be adversely affected by excessive anhydrite content. The exothermic hydration of clinker was accelerated by calcium sulfate at the beginning, but the rate declined as the process progressed. Sufficient sulfur supply can enhance the hydration of ye’elimite, thereby increasing the AFt content in the hydration product. The mass loss of the hydration product is mainly caused by the dehydration of ettringite, monosulfoaluminate, and AH_3_. In addition, the Na element in the RCFA hydration product is mainly present in monosulfoaluminate and unhydrated cement particles.

## 1. Introduction

Calcium ferroaluminate cement (CFA) has shown great potential in marine engineering due to its excellent durability, but the high cost of this cement has affected its large-scale promotion and application [1,2,3]. Red mud, as a high-iron industrial solid waste, has attracted the interest of researchers in the preparation of cement clinker because its chemical composition is like that of raw cement materials [4,5]. Red mud-based CFA (RCFA) has been shown to differ from CFA in composition and hydration due to the high amount of alkali (Na_2_O/K_2_O) in red mud [6]. For example, Na ions can replace the lattice sites of Ca ions in C_4_A_3_$, thereby promoting the hydration of minerals.

The performance of CFA is mainly determined by the hydration reaction between clinker and calcium sulfate. By adjusting the calcium sulfate content, cement with different properties can be produced [7,8,9,10,11,12,13,14,15,16]. Calcium sulfate plays an indispensable role in the hydration process of CFA. For CSA, calcium sulfate in this system does not only play a role in regulating setting, but is deeply involved in the hydration process, regulating strength and expansion properties. With the shortage of natural gypsum resources, many studies currently focus on the effect of gypsum dosage on the hydration properties of CSA. Researchers believe that the addition of gypsum shortens the setting time of cement. An appropriate amount of gypsum can promote the hydration of C_4_A_3_$ and C_2_S and promote the formation of ettringite, which provides mechanical properties for the hardened cement paste. In addition, the hydration of the ferrite phase in the CFA will form Fe-containing ettringite. When the gypsum content in the hydration system is low, the ettingite will transform into CaAl-LDHs [17] and CaAlFe-LDHs [18,19,20], which are the common monosulfoaluminates. As the amount of gypsum increases, the number of C-S-H decreases, while the number of AFt increases accordingly [21,22].

In the case of RCFA, some sulfates are formed due to the presence of alkali in the red mud, and whether this change has any effect on the selection of calcium sulfate is unknown. Due to the different dissolution characteristics of calcium sulfate, there are significant differences in the hydration rate of cement and the formation of hydration products, but they all affect overall hydration by participating in the generation of ettringite [23]. Common types of calcium sulfate include gypsum, hemihydrate, anhydrite, industrial by-product desulfurization gypsum, etc. The addition of calcium sulfate changes the hydration kinetics of C_4_A_3_$, thereby changing the ratio of ettringite to monosulfoaluminate in the hydration product. More reactive calcium sulfates, such as gypsum or calcium hemihydrate, are more effective than anhydrite in promoting early terminal dissolution [13,15,16,24,25,26,27,28]. The dissolution kinetics of anhydrite are much slower compared to gypsum or hemihydrate, which results in an insufficient supply of calcium and sulfate ions and is not conducive to the formation of ettringite. Winnefeld shows that the presence of gypsum also accelerated the dissolution of anhydrite and that replacing part of the anhydrite with gypsum could improve the hydration kinetics and the early and late compressive strengths of CSA [25]. García-Maté indicates that the dissolution rate of the different studied sulfate sources is the key factor for the hydration of CSA [27].

It can be seen from the current data that calcium sulfate is the key to solving the hydration rate and physical properties of high-alkali RCFA cement. However, ferroaluminate cement prepared from red mud has very high Na characteristics. Exploring how to use calcium sulfate to regulate the hydration of high-alkali red mud-based ferroaluminate cement is key to the efficient utilization of red mud. The data available regarding the impact of the types and dosage of calcium sulfate on the hydration kinetics and strength development of red mud-based ferroaluminate cement remain rather limited. To tackle the current research gaps and gain an in-depth understanding of the effect of calcium sulfate types on the hydration of RCFA, more detailed work is needed.

The objective of this work is to reveal the influence of calcium sulfate on the hydration rate of clinker by adding different types and amounts of calcium sulfate to clinker, evaluating the influence of different sulfate on the mechanical properties of cement and the phase assemblages of hydration products. Finally, the morphology and properties of the hydration products are discussed based on different dosages. This paper provides a theoretical basis for calcium sulfate to regulate the hydration of RCFA and provides technical support for the application of high-alkali red mud.

## 2. Materials and Methods

### 2.1. Materials

Raw materials used for the synthesis of RCFA clinkers were analytical-grade CaCO_3_, SiO_2_, Al_2_O_3_, CaSO_4_·2H_2_O, and red mud produced in Long Kou, China. Red mud was used to provide all the Fe_2_O_3_ in cement raw meal, and its chemical composition is shown in Table 1. The calcium sulfate samples used in this paper were all analytically pure and produced by Sinopharm Reagent Co., Ltd., (Shanghai, China) of which the purity of gypsum was 99.0% and the purity of anhydrite was 97.0%.

### 2.2. Sample Preparation

The theoretic phase composition of RCFA clinkers is shown in Table 2. The amount of red mud in the raw materials was 24.27 wt.%. The raw meals were homogenized in a planetary ball mill (YXQM-4L) at a speed of 400 r/min for 1 h. Subsequently, the raw meal was mixed with 7 wt.% water and compressed into Φ45 mm × 3 mm disk models under a pressure of 15 MPa. The cakes were dried at 105 °C for 2 h in an oven to eliminate retained water, followed by sintering the dried disks at 1175 °C in a resistance furnace for 30 min. After that, the sintered material was removed from the furnace and rapidly cooled with a fan to prevent clinker mineral phase decomposition. It was then ground in a ball mill to pass through a 75 μm sieve and mixed with gypsum or anhydrite (chemical grade) to produce RCFA. The different dosage of calcium sulfate in the clinker is shown in Table 3.

**Figure 1:**
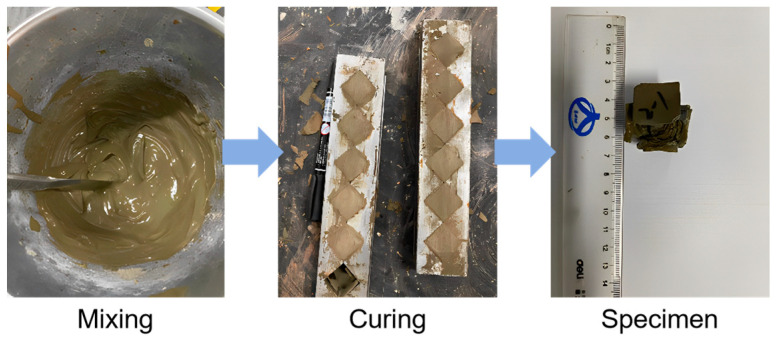
Photos of specimens.

All the cement pastes were prepared with a water-to-cement ratio (w/c) of 0.4, and then the obtained paste was placed into a standard curing room with 98% relative humidity at 20 °C to the required aging time. Photos of specimens in the molding process are shown in Figure 1.

### 2.3. Methodology

#### 2.3.1. Mechanical Properties

The compressive strength of hardened cement pastes samples (20 mm × 20 mm × 20 mm) was measured by using the servo-hydraulic test machine (MTS-CMT5504, MTS Industrial Systems (China) Co., Ltd., ShenZhen, China) with a maximum capacity of 100 kN, and the loading rate was 0.5 MPa·s^−1^ [29]. The average value of compressive strength was calculated from three replicate samples at designated ages (1, 3, and 28 days, respectively).

#### 2.3.2. Isothermal Calorimetry

With the help of an eight-channel isothermal calorimeter (TAM Air, TA Instruments Ltd., New Castle, DE, USA), the samples were analyzed for heat release by the internal stirring method. Two grams of cement were mixed with one gram of water, and the heat flow was recorded for 24 h at 20 °C. To obtain the cumulative heat, the heat flow curves were integrated. According to the designed water–ash ratio, the appropriate amount of sample and water were weighed into an ampoule and a water gun, respectively; then the water gun and the ampoule were put into the instrument to equilibrate for 6 h at a temperature of 25 °C; after equilibrating, the water was added into the ampoule and stirred for 1 min, and the hydration exothermic curves were determined during the required reaction time.

#### 2.3.3. X-ray Powder Diffraction

All clinker specimens were finely ground into powder for laboratory X-ray powder diffraction analysis. A phase assemblage analysis was conducted using a (D8-Advance, Bruker AXS GmbH, Karlsruhe, Germany) X-ray diffractometer from Bruker with a Lynx Eye detector, utilizing Cu Ka radiation (λ = 0.15 nm) at 40 kV and 40 mA. The comprehensive measurements spanned a two-theta range from 5° to 50° in 0.02° increments. A scan speed of 2°/min was implemented to ensure precise quantification. The identification of clinker minerals was implemented by analysis using Jade v6 software. Rietveld refinement quantitative phase analyses were performed using TOPAS v6 software. In the quantitative phase analysis, the crystal structure information of each phase is shown in Table 4.

To study the influence of calcium sulfate on the hydration products of RCFA, a qualitative XRD analysis was performed on the paste of RCFA mixed with different calcium sulfate contents after 3 days and 28 days of hydration. The water-to-cement ratio (w/c) of cement paste was 0.4. The cement paste was placed in centrifuge tubes filled with isopropanol. After 24 h, the isopropanol was replaced, and cement hydration was terminated by allowing the samples to stand for 24 h. The samples were then dried in a vacuum drying oven at 40 °C and 0.8 MPa of vacuum pressure. After grinding and passing through a 200-mesh sieve, the samples were subjected to testing.

#### 2.3.4. Differential Scanning Calorimetry–Thermogravimetric Analysis

Differential scanning calorimetry–thermogravimetric analysis (DSC-TG) can characterize the physical and chemical reactions and mass fraction changes of the hydrated samples. In this paper, a STA449F3 synchronous thermal analyzer produced by NETZSCH, Germany was used for testing. The protective gas was air and N_2,_ and the gas flows were 30 mL·min^−1^ and 50 mL·min^−1^, separately. The test temperature was from 25 °C to 1000 °C, at a heating rate of 10 °C/min under air atmosphere.

#### 2.3.5. Morphology Analysis

The morphological feature and elemental composition of the samples were characterized by a Carl Zeiss GEMINI 360 scanning electron microscope and an Oxford X-Max energy dispersive spectrometer, respectively. The test was performed at a voltage of 20 kV, a current of 20 μA, and a working distance of 11.5 mm. The counting point of EDS was greater than 5000 cps, and the data acquisition time was 30–60 s.

## 3. Results and Discussion

### 3.1. The Phase Analysis of RCFA Clinker

**Figure 2:**
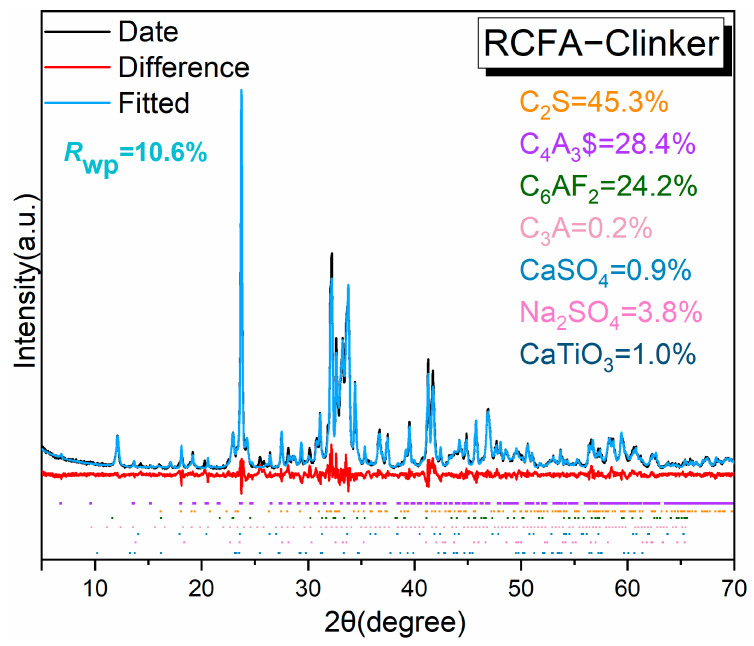
XRD pattern of RCFA clinker.

XRD was employed to analyze the phase composition of clinker specimens. The clinkers without and with C$, burned at 1175 °C, are shown in Figure 2. The main minerals of the clinker included belite (C_2_S), ye’elimite (C_4_A_3_$), and ferrite phase (C_6_AF_2_). There are trace minerals such as C_3_A, sulfate, and inert mineral perovskite. Due to the high content of red mud, which introduces additional Na_2_O, Canbek suggests that Na_2_O has a strong affinity for sulfate during the clinker-burning process, leading to the formation of sodium sulfate in the clinker. This formation can cause a discrepancy between the actual content of the main mineral ye’elimite (C_4_A_3_$) and its theoretical value, which indirectly promotes the formation of the aluminum phase (C_3_A). Additionally, the peak of the ferrite phase is close to the characteristic peak of C_6_AF_2_ at approximately 12.0°.

The Topas v6 software was used for the quantitative analysis of the clinker, and its Rwp value was 10.6%. This value falls within an acceptable range, making the data dependable [30,31,32]. The quantitative results are presented in Figure 2. The ferrite phase content is slightly higher than the design value, but the primary mineral phase C_4_A_3_$ is lower than the target value due to the presence of alkalis. The refined data indicate the presence of sodium sulfate and C_3_A in the clinker, which deviates from the designed mineral composition. Additionally, the clinker contains 0.9% calcium sulfate by weight. This finding supports the hypothesis that sodium has a strong affinity for additional sulfate to form sodium sulfate. The calcium in the raw materials contributes to the formation of belite and high-calcium ferrite phases, resulting in their contents being higher than intended.

### 3.2. Compressive Strength

**Figure 3:**
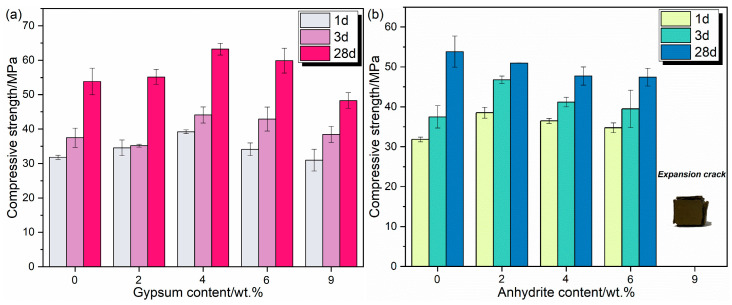
Compressive strength of RCFA with different kinds of calcium sulfate dosage: (**a**) Gypsum, (**b**) Anhydrite.

After mixing the clinker with different types of calcium sulfate for the preparation of the cement paste, molding experiments were carried out under the condition of a water–cement (w/c) ratio of 0.4, and their 1 d, 3 d, and 28 d compressive strengths were determined, respectively; the results are shown in Figure 3.

The compressive strength of RCFA specimens with gypsum at different curing ages is shown in Figure 3a. It is evident that the compressive strength generally improves across all age groups (1 d, 3 d, and 28 d) as the gypsum content increases. The addition of gypsum appears to have a favorable impact on the compressive strength of the cement paste, particularly when comparing the 0 wt.% gypsum level to others. However, the improvement becomes less noticeable once the gypsum content exceeds 4 wt.%. This observation can be explained by the fact that at low gypsum concentrations, the dominant hydration product of C_4_A_3_$ is the AFm phase. As the gypsum content increases, sufficient availability of gypsum promotes the formation of AFt and AH_3_, leading to enhanced strength. At 4% gypsum content, the cement paste has a 3 d compressive strength of 39.1 MPa and a 28 d compressive strength of 63.2 MPa, an increase of 61.6% over 3 d, indicating good early strength and good mid- and late-stage strength development at this dosage.

Despite this, an overabundance of gypsum reacts with AH_3_ to generate a significant amount of AFt, which compromises the dense structure of the cement paste, thus reducing its strength. Furthermore, the rate of increase in cement compressive strength at 28 d exceeds that at 3 d, indicating that the solubility of gypsum is relatively low, allowing it to dissolve gradually during the dehydration process. This gradual dissolution enables the reaction with non-hydrated C_4_A_3_$ or existing AH_3_ to produce AFt, which enhances strength over time.

As depicted in Figure 3b, the early compressive strength of cement paste initially increases and then decreases as the anhydrite content increases, with the highest early strength observed at a 2 wt.% anhydrite concentration. This trend parallels the behavior observed with gypsum. However, the expansion crack in the 9 wt.% anhydrite sample indicates that excessive amounts of anhydrite may lead to cracking issues, likely due to increased hydration reactions causing volume expansion. This is attributed to the solubility of anhydrite, which, when present in excessive amounts, initiates a rapid reaction between C_4_A_3_$ and anhydrite, resulting in the extensive formation of AFt that induces cracks and leads to a reduction in strength. However, previous studies believed that the addition of anhydrite had little effect on CSA, and the rapid dissolution of gypsum promoted the formation of AFt [17]. The strength growth rate at 28 d for the cement paste is subdued, and its compressive strength progressively diminishes with increasing anhydrite content. This is due to the significant formation of AFt during the early stages of cement hydration, which causes slight expansion and results in a less compact internal structure. Consequently, this hampers the strength development in later stages. This also explains why the late-stage strength of cement paste with anhydrite is inferior to that of paste with gypsum at equivalent content levels. In conclusion, both gypsum and anhydrite contribute positively to the compressive strength of cement paste, with gypsum having a stronger impact. However, excessive amounts of anhydrite may cause expansion cracks, highlighting the importance of maintaining optimal levels of additives in the cement paste formulation.

### 3.3. Hydration Heat

**Figure 4:**
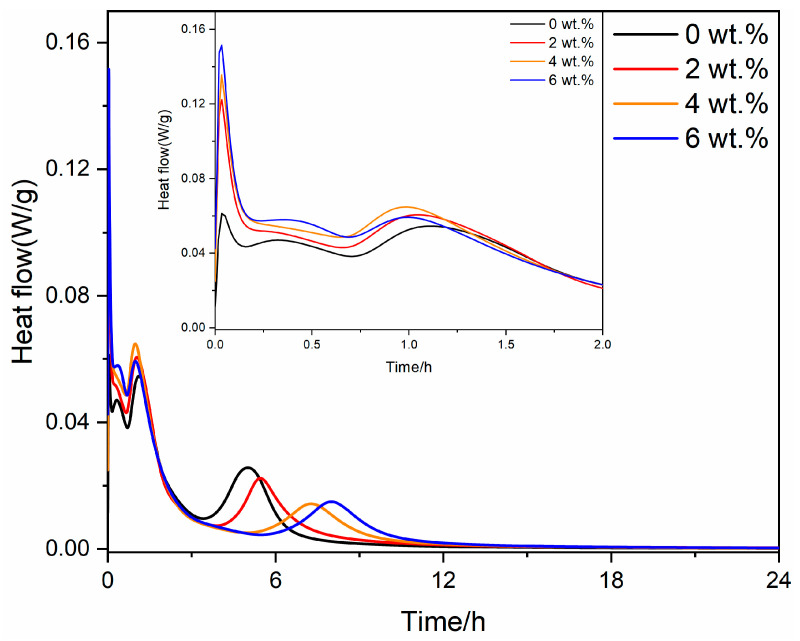
Heat release of RCFA with different dosages of gypsum.

**Figure 5:**
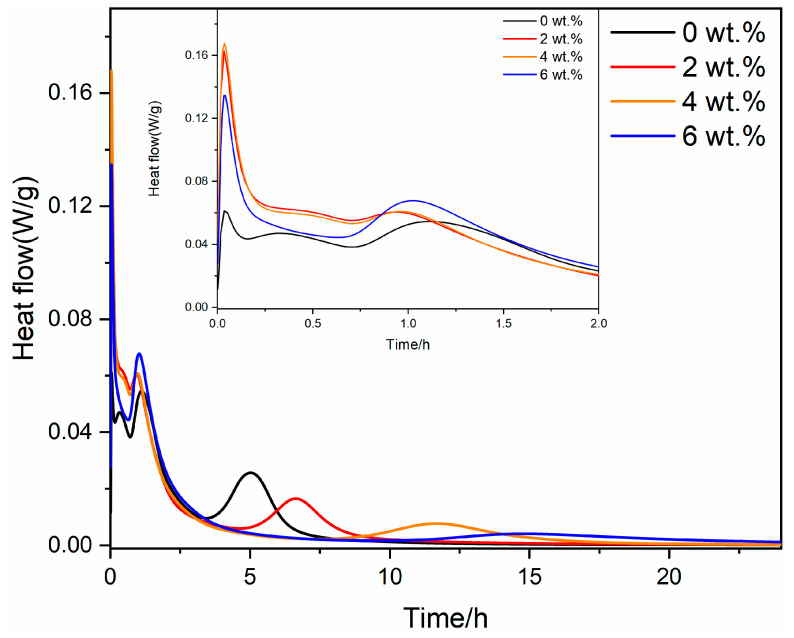
Heat release of RCFA with different dosages of anhydrite.

To investigate the influence of gypsum on the hydration process of RCFA clinker, a hydration calorimetry analysis was conducted on RCFA with different gypsum contents. The results are shown in Figure 4 and Figure 5.

Upon examining Figure 4, it is evident that each clinker specimen exhibits four distinct peaks in the rate of hydration heat release. Incorporating gypsum results in an escalation of the peak values for the first three hydration events, while a decline is observed in the peak value associated with the fourth event. The initial sharp rise in heat flow for all samples signifies a vigorous hydration reaction upon exposure to water. Furthermore, as the gypsum content increases, the heat flow curve shifts to the right, indicating a delay in the hydration reaction and a widening of the peaks. The heat flow declines rapidly for all samples after the initial peak, signaling the onset of a slower hydration stage. This decline is more gradual for samples with greater gypsum content, demonstrating the extended hydration process induced by the presence of gypsum. Eventually, the heat flow stabilizes for all samples, marking the attainment of equilibrium. The time required to achieve this steady state varies according to the gypsum content, with higher gypsum levels necessitating a longer duration to reach equilibrium. These findings imply that gypsum accelerates the early stages of clinker hydration heat release, albeit at the cost of a decreased rate during subsequent stages.

Figure 5 illustrates the rate of the hydration exothermic reaction for RCFA clinker following the incorporation of anhydrite. An analysis of Figure 5 reveals that, in the absence of anhydrite, the clinker hydration exothermic rate curves display four distinctive peaks. Upon the introduction of anhydrite, the second exothermic peak is notably reduced, whereas the first and third peaks experience an increase in magnitude. Conversely, the intensity of the fourth exothermic peak is diminished. Not only do the first and third peaks see an increase in their values, but the onset of the fourth peak is also delayed, and its shape becomes more diffuse. This suggests that the addition of anhydrite accelerates the initial exothermic rate of clinker hydration, resulting in a subsequent deceleration of the exothermic rate, prolonging the duration of heat release. Based on Figure 4, it is evident that at the same dosage, the anhydrite sample exhibits a higher peak value for the first hydration exothermic peak. This may be the reason for the expansion and rupture of the anhydrite sample.

### 3.4. Analysis of Hydration Products

#### 3.4.1. XRD Analysis

**Figure 6:**
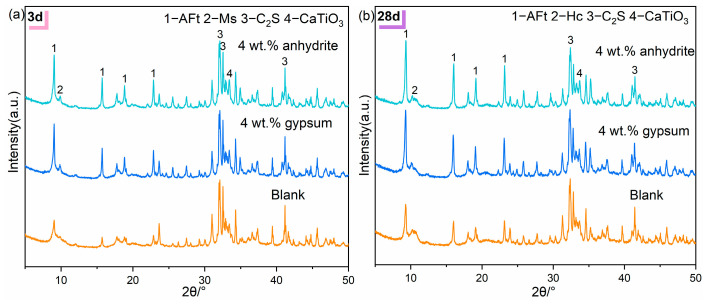
XRD pattern of hydrated products of RCFA: (**a**) 3 d, (**b**) 28 d.

Figure 6a,b show the XRD patterns of the hydration products of RCFA without gypsum and with 4 wt.% gypsum and anhydrite, respectively.

As can be seen in Figure 6, the hydration products of all the samples are AFt, AFm as well as non-hydrated C_2_S and CaTiO_3_ with no hydration activity. The gradual enhancement of the AFt diffraction peaks in the hydration products after gypsum doping is attributed to the fact that gypsum doping promotes the conversion of AFm to AFt. With the addition of calcium sulfate, the peak intensity of hydration product AFt increases significantly at 3 d, indicating that the incorporation of gypsum is conducive to the formation of ettringite. This trend is also reflected in the 28 d hydration product.

The main hydration reaction that occurs when RCFA is exposed to water is as follows:

3CaO·3Al_2_O_3_·CaSO_4_ + 2(CaSO_4_·2H_2_O) + 34H_2_O→3CaO·Al_2_O_3_·3CaSO_4_·32H_2_O + 2(Al_2_O_3_·3H_2_O)

When the gypsum content in cement is insufficient, the hydration reaction occurs mainly as follows:

3CaO·3Al_2_O_3_·CaSO_4_ + 18H_2_O→3CaO·3Al_2_O_3_·CaSO_4_·12H_2_O + 2(Al_2_O_3_·3H_2_O)

3CaO·3Al_2_O_3_·CaSO_4_·32H_2_O→3CaO·3Al_2_O_3_·CaSO_4_·12H_2_O + 2(CaSO_4_·2H_2_O) + 16H_2_O

C_6_AF_2_ + 3 CaSO_4_·2H_2_O +35 H_2_O→3CaO[xAl_2_O_3_·(1 − x)Fe_2_O_3_]·3CaSO_4_·32H_2_O + 3Ca(OH)_2_ + (2 + 2x) FH_3_ + (2 − 2x) AH_3_

C_2_S + 2H→C-S-H + CH

Mixing gypsum in cement, with the increase in gypsum dosing, can promote the conversion of AFm to AFt, and the reaction process is shown below:

3CaO·3Al_2_O_3_·CaSO_4_·12H_2_O + 2(CaSO_4_·2H_2_O) + 16H_2_O→3CaO·Al_2_O_3_·3CaSO_4_·32H_2_O

At a hydration age of 3 d, the AFt diffraction peak intensity of the calcium sulfate group is notably higher than that of the control group, indicating a greater content of AFt. The peak values for the calcium sulfate group and the blank group do not differ significantly, with some AFm evident. Compared to cement samples with 4 wt.% anhydrite, those with 4 wt.% gypsum show higher AFt diffraction peaks in their hydration products. This phenomenon could be attributed to the low solubility of gypsum combined with its rapid dissolution rate, which continually supplies a sulfur source for the formation of AFt. Due to the high content of red mud, the diffraction peak is located at about 10° and an iron-containing AFm phase is formed, namely CaFe and CaAlFe layered double hydroxides (LDHs) [19]. This may be due to the transformation of 3CaO[xAl_2_O_3_·(1 − x)Fe_2_O_3_]·3CaSO_4_·32H_2_O. Furthermore, by 28 d of hydration, the AFm diffraction peak, which is observable at 3 d, vanishes, giving rise to a hemicarbonate, which may be due to the ongoing hydration process being affected by CO_2_.

#### 3.4.2. DSC-TG Analysis

**Figure 7:**
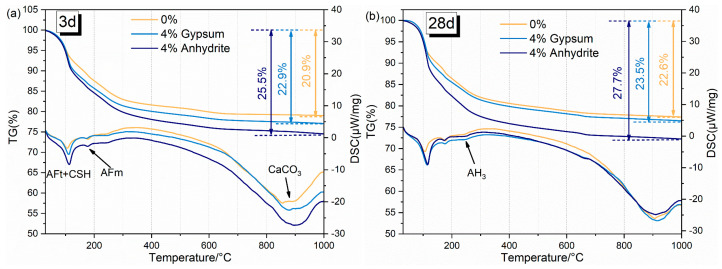
DSCTG of hydrated products of RCFA: (**a**) 3 d, (**b**) 28 d.

Amorphous phases such as AH_3_ were difficult to reflect in the XRD test, so the hydration products of RCFA were further subjected to DSC-TG analysis, the results of which are shown in Figure 7, where Figure 7a,b show the DSC-TG curves of the hydration products of RCFA hydrated for 3 d and 28 d, respectively.

The DSC-TG curve depicted in Figure 7 demonstrates that, as the temperature increases, the mass of the hydration products derived from RCFA progressively decreases, with the bulk of the mass loss concentrated in the range of 100–300 °C. This mass loss is predominantly attributed to the dehydration of AFt, AFm, and AH_3_. The endothermic peak associated with the dehydration of AFt is observed at approximately 100 °C, corresponding to the loss of 20 moles of bound water per mole of AFt. The endothermic peak for the dehydration of AFm is situated around 190 °C, whereas the endothermic peak for AH_3_ dehydration occurs at roughly 260 °C, involving the loss of 3 moles of bound water per mole of AH_3_ [33]. The incorporation of gypsum into the cement matrix facilitates the formation of AFt and enables the expulsion of a greater amount of bound water at elevated temperatures. Consequently, the mass loss of the hydration products of cement augmented with calcium sulfate surpasses that of cement devoid of gypsum. The curve representing the hydration products at 3 days further highlights that the gypsum group displays a more pronounced endothermic peak within the 100–300 °C temperature range, whereas the anhydrite group manifests a significantly greater weight loss. This disparity in behavior can be attributed to the differing solubilities of gypsum and anhydrite, coupled with the higher SO_3_ content in anhydrite. These factors culminate in a higher AFt content within the cement hydration products, leading to a more substantial mass loss during thermal analysis. Moreover, as the hydration age increases, the mass loss of each group gradually increases, which is consistent with the XRD results of the hydration products in the previous paper.

#### 3.4.3. SEM Analysis

**Figure 8:**
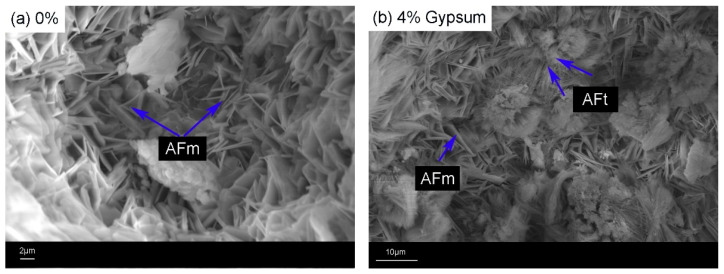
Morphology of hydrated products of RCFA.

The micromorphology of the hydration products and the distribution of alkali in the hydration products were analyzed by SEM-EDS, and the results are shown in Figure 8.

**Figure 9:**
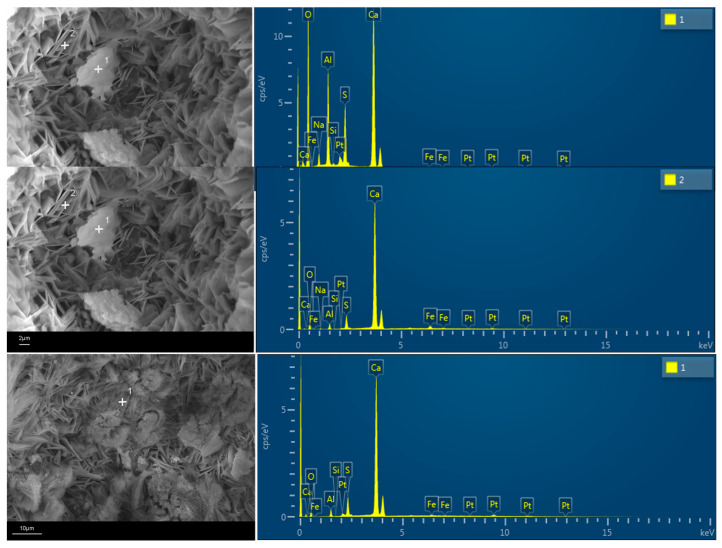
EDS of hydrated products of RCFA: +1 and +2 mean element confirmation of the target point.

The SEM image of the hydration products in Figure 8 shows that in the sample without gypsum, there is an abundance of layered AFm, a small amount of needle-like AFt and non-hydrated cement particles that are interwoven to provide strength to the cement paste. In the sample with added gypsum, there is an increase in needle-like AFt and a decrease in layered AFm, indicating that the addition of gypsum promotes the transformation of AFm to AFt. The elemental analysis results in Figure 9 show that sodium (Na) element is mainly distributed in AFm, with a small amount also present in the non-hydrated cement particles, while no sodium is found in AFt. In addition, Fe was found in the flaky AFm material, indicating that CaAlFe-LDHs might be formed, which is consistent with the previous analysis (Section 3.4.1).

## 4. Conclusions

The main issue of this paper is the effect of the calcium sulfate type on the hydration of red mud-based ferroaluminate cement (RCFA) clinker. Based on the above experimental results, the following conclusions are drawn:

(1) XRD analysis reveals the presence of the main minerals like C_2_S, C_4_A_3_$, and ferrite phase (C_6_AF_2_) and trace minerals like sulfate in the RCFA clinker. The cement paste is 61.6% stronger when 4% gypsum is added, with a 3 d compressive strength of 39.1 MPa and a 28 d compressive strength of 63.2 MPa. Excessive anhydrite content can precipitate deleterious effects on the properties of cement paste.
(2) RCFA clinker is heated by both gypsum and anhydrite during the initial phase of hydration, but anhydrite contributes more to the initial stage. As the gypsum content increases, the longer it takes to reach equilibrium in the hydration heat release curve.
(3) The RCFA main hydration products are AFt and AFm, along with non-hydrated C_2_S and CaTiO_3_. A sufficient sulfur source can promote the hydration of C_4_A_3_$, thereby increasing the AFt in the hydration product.
(4) The mass loss of hydration products from RCFA is mainly due to the dehydration of AFt, AFm, and AH_3_. The calcium sulfate group lost more mass than the blank group. AFm and non-hydrated cement particles contained most of the Na in the hardened cement paste.

The effects of gypsum and anhydrite RCFA are different from CSA cement. Gypsum has shown a better ability to regulate the heat of cement hydration and promote the development of strength in the middle and late stages during the hydration of RCFA clinker. Therefore, according to specific requirements, the properties of RCFA cement clinker can be properly regulated by gypsum, thereby optimizing the production cost in practical applications.

## Figures and Tables

**Table 1:** Chemical composition of red mud (wt.%).

Chemical Composition	CaO	SiO_2_	Al_2_O_3_	Fe_2_O_3_	Na_2_O	TiO_2_	K_2_O	Others
Red mud	3.56	15.33	22.50	33.56	8.68	6.45	0.06	9.86

**Table 2:** Phase and chemical composition of RCFA clinker (wt.%).

Sample	Mineral Composition				Chemical Composition					
RCFA	C_4_A_3_$	C_2_F	C_2_S	CaSO_4_	CaO	SiO_2_	Al_2_O_3_	Fe_2_O_3_	SO_3_	Na_2_O
	35	20	35	10	48.0	12.2	17.6	11.8	10.5	2.8

**Table 3:** Mixture proportions of RCFA (wt.%).

Type	Content			
Gypsum	2	4	6	9
Anhydrite	2	4	6	9

**Table 4:** The structural details of the main phases.

Phase	Space Group	ICSD Codes
c-C_4_A_3_$	I-43 m	9560
o-C_4_A_3_$	Pcc2	80361
β-C_2_S	P21/n	79553
C_6_AF_2_	Ibm2	1000040
C_3_A	Pa-3	1841
Na_2_SO_4_	Fddd-70	1903926
CaSO_4_	Amma	40043
CaTiO_3_	Pbnm	163528
AFt	P31c	155395
AFm	R-3	24461

## Data Availability

A COD card to a Crystallography Open Database.

## Nomenclature

C_4_A_3_$	3CaO·Al_2_O_3_·CaSO_4_
C_2_S	2CaO·SiO_2_
AFt	3CaO·Al_2_O_3_·3CaSO_4_·32H_2_O
AFm	3CaO·3Al_2_O_3_·CaSO_4_·12H_2_O
AH_3_	Al_2_O_3_·3H_2_O(gel)
FH_3_	Fe_2_O_3_·3H_2_O(gel)
CH	Ca(OH)_2_
